# Reflections on How Wheat Flour Lipids Impact the Viscoelastic Properties of Gluten Proteins

**DOI:** 10.1111/1541-4337.70382

**Published:** 2026-01-14

**Authors:** Gamze Yazar, Jozef L. Kokini

**Affiliations:** ^1^ Department of Animal, Veterinary and Food Sciences University of Idaho Moscow Idaho USA; ^2^ Food Science Department Purdue University West Lafayette Indiana USA

**Keywords:** endogenous wheat lipids, gluten network, molecular interactions, rheology

## Abstract

Endogenous wheat flour lipids are known to play an important role in baked product quality, even though they constitute a minor fraction of wheat flour composition. They interact with gluten proteins throughout the different stages of dough processing and therefore contribute to specific quality traits in baked products. Polar non‐starch lipids interact with gliadins, whereas both polar and nonpolar non‐starch lipids interact with glutenins. These interactions control the hydration dynamics of the gluten proteins during mixing, contribute to the gluten network strength, prevent coalescence of the gas cells during fermentation, and affect the gluten denaturation and starch gelatinization temperatures during baking. The chemical backgrounds of these interactions have been of interest. The aim of this review is to bring an in‐depth evaluation of how the interactions between the endogenous wheat flour lipids and gluten proteins affect the mechanical properties of the gluten network.

## 1 Introduction

Wheat flour contains around 1.5%–3.0% of lipids (Janssen et al. 2018; Melis and Delcour 2020; Yazar et al. 2022; Yazar, Kokini, et al. 2024). The lipids in wheat flour are often referred to as endogenous lipids (Pareyt et al. 2011; Yazar, Kokini, et al. 2024). About 35%–45% of endogenous lipids occur in the endosperm, 30%–36% in the germ, 25%–29% in the aleurone, and only a minor portion (<4%) in the pericarp (Melis and Delcour 2020). Lipids in the endosperm are typically classified as starch lipids, which occur inside starch granules, or as non‐starch lipids. The latter are further subdivided into free lipids subclasses (FLs) and bound lipids (BLs) based on their sequential extractability with nonpolar (e.g., hexane) and polar (i.e., water saturated butanol [WSB]) solvents, respectively. The most abundant nonpolar lipids in wheat flour are triacylglycerols (TAGs) and free fatty acids (FFA). Glycolipids and phospholipids constitute the majority of polar lipids (Pareyt et al. 2011; Gerits et al. 2013; Janssen et al. 2018; Yazar et al. 2022; Yazar and Rosell 2023). Although endogenous lipids constitute a small percentage in wheat flour composition, they play an important role in breadmaking. Due to their amphipathic nature (hydrophilic and hydrophobic groups present), endogenous lipids have the ability to associate with gluten proteins and starch (Melis and Delcour 2020), leading to a variety of beneficial properties from dough processing to end‐product storage, and thus allowing a great deal of flexibility in cereal product development and giving the product developer the ability to obtain a wide variety of textures (Pareyt et al. 2011).

Earlier studies have shown through the use of spectroscopy and imaging techniques that endogenous lipids interacted with gluten proteins in specific ways (Hess and Mahl 1954; Grosskreutz 1961; Hoseney et al. 1970; Wehrli and Pomeranz 1970a, 1970b; McCann et al. 2009). To further explore these interaction mechanisms, the distribution of lipids during breadmaking and storage and the interactions between gluten proteins and lipids in wheat flour dough have been studied through wet chemistry methods (Li et al. 2004; Sroan and MacRitchie 2009; Gerits et al. 2014; Janssen et al. 2018). The traditionally used methods included using different quality wheat flours for lipid characterization and breadmaking, defatting the wheat flour and then reconstituting it with the extracted lipids, and adding wheat lipids or lipids from other sources to regular or defatted wheat flour (Melis and Delcour 2020). Due to constituting a tiny percentage in wheat flour, the impact of lipids on unique dough viscoelastic structure is mostly neglected. However, the machinability and moldability of the dough strongly depend on this unique viscoelastic response (Yazar 2023a). On the other hand, most of the studies evaluating the interactions among gluten proteins and wheat lipids were conducted in a wheat flour dough system, where starch is also involved as a major fraction. Therefore, there is a gap in literature evaluating the direct impact of endogenous lipid–gluten interactions on the mechanical properties of the gluten network.

This review is novel as it focuses on the linear and nonlinear viscoelastic properties of gluten and its main fractions, gliadin and glutenin, both in the presence and absence of endogenous wheat lipids to unravel the contribution of endogenous lipids to the viscoelastic nature of the gluten network. The molecular insights gained from these empirical and fundamental rheological measurements can help understand the mechanically driven molecular interactions occurring between gluten proteins and endogenous lipids during dough processing. Considering gluten proteins are responsible for the unique viscoelastic properties of wheat flour dough, leading to desired traits in baked products, understanding the impact of gluten–lipid interactions on the viscoelastic properties of the gluten network sheds light on how these molecular interactions can affect end‐product quality.

## 2 Gluten–Lipid Interactions

### 2.1 Models Describing the Interaction Mechanisms

**FIGURE 1:**
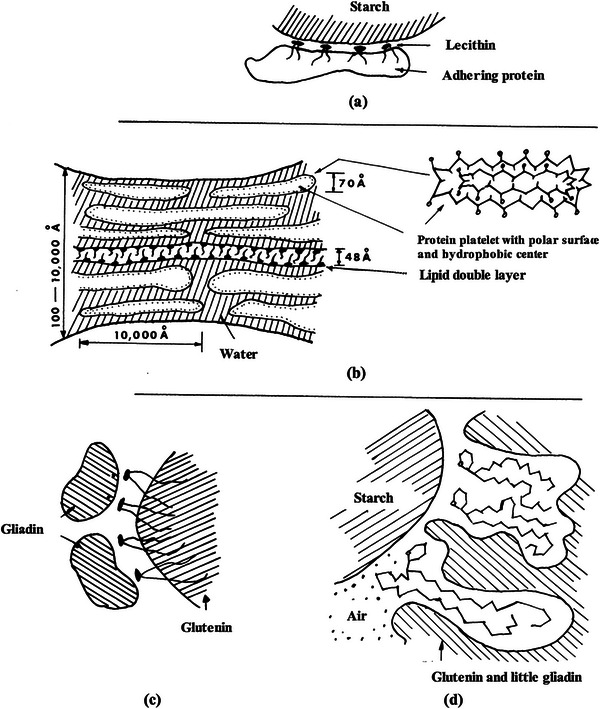
Models proposed to describe the interactions between gluten proteins and endogenous lipids: (a) starch–lipid‐adhesive protein interaction model by Hess and Mahl (1954), (b) the lipoprotein model by Grosskreutz (1961), (c) gliadin–glycolipid–glutenin complex model by Hoseney et al. (1970), (d) starch–glycolipid–gluten complex model by Wehrli and Pomeranz (1970a, 1970b). *Source*: Reproduced from Pomeranz and Chung (1978) with the permission from the publisher.

Several models have been proposed so far to explain the nature of protein–lipid interactions in wheat flour dough (Pomeranz and Chung 1978; Yazar and Otles 2012). Olcott and Mecham (1947) found that lipids were strongly bound to glutenin to form a “lipoglutenin” complex. Hess and Mahl (1954) proposed a model (Figure 1a), where the adhesive protein is bound to starch through a lecithin layer to a protein–lipid–starch complex in wheat flour dough. Grosskreutz (1961) used x‐ray spectroscopy and electron microscopy to study wheat gluten structure at the molecular level. The result of these studies revealed that gluten proteins exist in platelets with a thickness of 70 Å and provided evidence of a phospholipid layer structure in developed gluten, resembling the well‐oriented bimolecular leaflets found in myelin (Grosskreutz 1961). Thus, Grosskreutz proposed the lipoprotein model seen in Figure 1b where the lipoprotein constituted around 2%–5% of the gluten structure. In this lipoprotein model, protein chains are bound to the outer edge of the phospholipid bimolecular leaflet array through salt‐like linkages between the acidic groups of the phospholipid and the basic protein groups. The hydrophobic side chains of the protein penetrate the lipid bimolecular leaflet, whereas the hydrophilic protein side chains face out to interact with other proteins or to absorb water. Hoseney et al. (1970) took these results one step further and suggested another model indicating that free polar lipids, principally glycolipids, were bound to gliadins by hydrophilic bonds and to glutenins through hydrophobic bonds (Figure 1c). The simultaneous binding of polar lipids to gluten proteins was suggested to contribute to the gas retaining ability of gluten network.

Following the model proposed by Hoseney et al. (1970), the interactions between glycolipids and wheat flour macromolecules were also studied by Wehrli and Pomeranz (1970a) using infrared spectroscopy and nuclear magnetic resonance (NMR). Infrared spectroscopy detected hydrogen bonds between glycolipids, gelatinized starch, and gluten proteins in addition to van der Waals bonds between glycolipids and gluten proteins. NMR spectra on the other hand suggested hydrophobic interactions between glycolipids and glutenin. Wehrli and Pomeranz (1970b) also used autoradiography to monitor the distribution of tritium‐labeled glycolipids in dough and bread.

The results revealed that glycolipids were mainly associated with gluten proteins and to a limited extent with the surface of starch granules in dough. On the other hand, a complex between glycolipids and starch resulted, suggesting a considerable interaction occurring between glycolipids and starch during gelatinization. The results obtained from these studies (Wehrli and Pomeranz 1970a, 1970b) led to a new model (Figure 1d) describing the mechanism behind the contribution of glycolipids to loaf volume. According to this model, glycolipids interact both with the gelatinizing starch and denaturing gluten proteins during baking, and thus, improve the gas‐retention by sealing the gas into the protein–lipid–starch complex cells (Pomeranz and Chung 1978).

Other studies that also used physical techniques, particularly NMR spectroscopy and freeze‐fracture electron microscopy, revealed that lipid was retained within the gluten in a relatively nonspecific way and the interaction of protein and lipid in gluten might involve the physical entrapment of lipid in addition to polar or ionic bonding (McCann et al. 2009).

Besides the use of microscopy and spectroscopy techniques, the interactions of endogenous lipids with other wheat constituents and the role of lipids in dough processing were evaluated using rheological methods or baking tests on dough samples prepared by removing the lipids and/or reconstituting defatted flour with certain lipid fractions (Georgopoulos et al. 2006; Sroan and MacRitchie 2009; Yazar et al. 2022; Yazar, Kokini, et al. 2024).

### 2.2 Functionality of Gluten–Lipid Interactions During Dough Processing

Wheat lipids have been traditionally classified as internal starch lipids and non‐starch lipids, where non‐starch lipids have been further subdivided into free and BLs. In addition to this classification based on solvent extractability, wheat lipids have also been classified as polar and nonpolar lipids. The classification of wheat lipids and their functionality throughout the different steps of the breadmaking process were extensively reviewed by Pareyt et al. (2011). A summary of the functionality of endogenous wheat lipids in the processing of bakery products was provided by Yazar and Rosell (2023).

**FIGURE 2:**
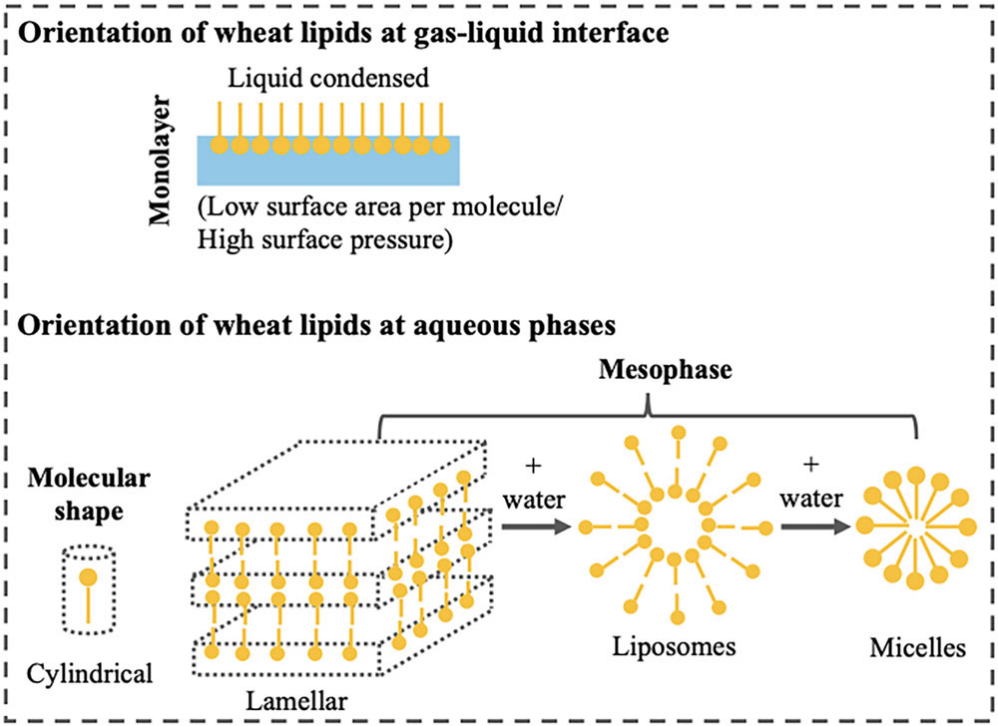
Orientation of endogenous wheat lipids at gas–liquid interface and at aqueous phases.

The functionality of internal starch lipids becomes evident during baking once starch gelatinization temperatures are reached (Yazar and Otles 2012; Melis and Delcour 2020). Internal starch lipids are unlikely to interact with other wheat components due to their location as they are buried inside the starch matrix. Therefore, starch lipids do not significantly affect loaf volume or crumb structure, but they may contribute to crumb texture by affecting the technological properties of starch (Carr et al. 1992; Melis and Delcour 2020). On the other hand, the functionality of endogenous non‐starch lipids starts to be significant at the first stage of the breadmaking process, mixing (Yazar et al. 2022), and continue until the early stages of baking (Sroan and MacRitchie 2009). Figure 2 shows the contribution of endogenous wheat lipids to gluten network development and gas cell stability. During dough development, an increase is observed in the BL level, whereas the level of free lipids is reported to decrease. The phenomenon that involves the redistribution of endogenous wheat non‐starch lipids is the result of lipid binding (Gerits et al. 2013, 2014). Besides, lipids are reported to be rubbed from the surface of the starch granules during dough development and they become trapped in or interact with gluten (Finnie et al. 2010).

The interactions between endogenous lipids and gluten were found to control the affinity of gluten proteins to water (Papantoniou et al. 2004) and thus to stabilize the consistency of gluten network during mixing (Yazar et al. 2022). As seen in Figure 2, polar lipids form highly ordered liquid–crystal phases (i.e., lamellar liquid–crystal phases) or mesophases (i.e., liposomes and micelles) when in contact with water (Gerits et al. 2014; Melis and Delcour 2020). The lamellar phase, consisting of lipid bilayer sheets separated by water (Carr et al. 1992), spontaneously forms small bilayer aggregates known as liposomes (Gerits et al. 2014).

**FIGURE 3:**
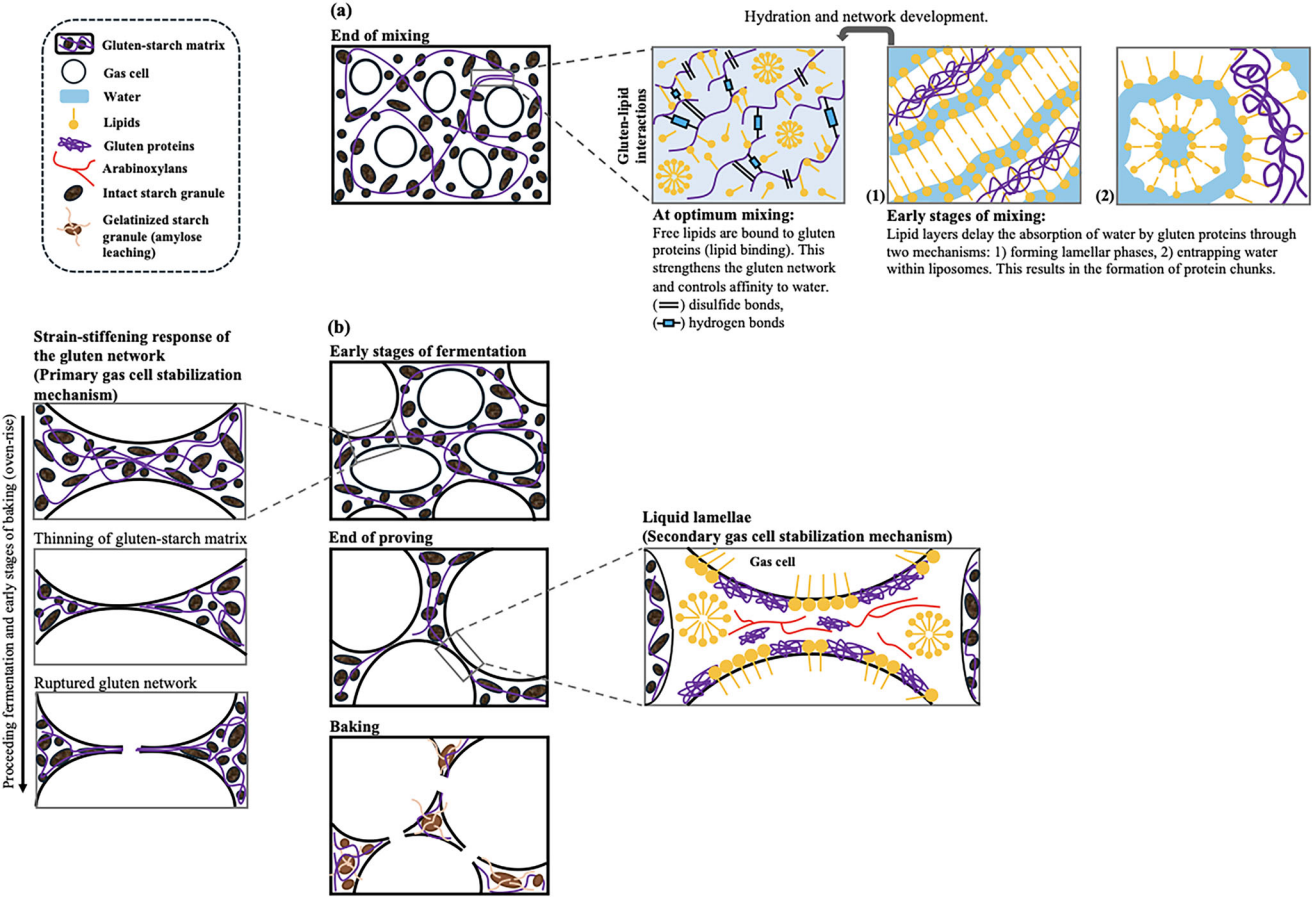
A schematical illustration of the structural transformation of dough during processing and the impact of endogenous wheat lipids on mechanical properties of dough: (a) Mixing [(1) formation of lamellar phases by lipids and (2) liposomes that entrap water during the early stages of mixing], (b) fermentation through baking: dough expansion and oven‐rise.

At the beginning of mixing, as flour particles meet water, the negatively charged hydrophilic head group of polar lipids may align themselves towards water, forming lamellar phases (Figure 3a‐1). Besides, free water can be entrapped by liposomes as shown in Figure 3a‐2. Both phenomena explain how lipids control the hydration dynamics of gluten proteins during dough mixing.

Gluten–lipid interactions in dough were also reported to contribute to the strength of the gluten network (Gerits et al. 2014; Yazar et al. 2022; Yazar and Rosell 2023). The gluten network determines how much air is incorporated in the dough (Sroan et al. 2009; Gerits et al. 2015). Lipids interacting with gluten indirectly stabilize gas cells and contribute to loaf volume (Gerits et al. 2014).

Besides the indirect effect on gas cell stabilization, endogenous polar lipids were reported to exert a direct effect during dough fermentation. Sroan and MacRitchie (2009) suggested a dual mechanism driven by the cooperative effect of the gluten network and the liquid lamellae surrounding the gas cells (Figure 3b). The biaxial extension of the gluten–starch matrix resulting from the expanding gas cells during proofing and oven‐rise causes thinning of the dough film surrounding the gas cells (Sroan et al. 2009). The extended gluten–starch matrix surrounding the gas cells is prevented from rupturing by the strain hardening or strain‐stiffening response of the gluten network (Figure 3b). With continued gas cell expansion, the strain‐stiffening behavior of gluten diminishes and the gluten network starts to deteriorate. The liquid lamellae around the gas cells, stabilized by the adsorbed surface‐active components such as proteins, polar lipids, and arabinoxylans, provide further gas cell stabilization during proofing and oven‐rise (Sroan and MacRitchie 2009). The lipid bilayers in the lamellar phase (Figure 2) are connected on the hydrocarbon chains located in the two sides of a lipid bilayer through the van der Waal's forces. These connections can break easily, causing the bilayers to separate and thus exposing the internal hydrophobic surface to interact with air (Carr et al. 1992; Gerits et al. 2014; Melis and Delcour 2020). This is how polar lipids are considered to align themselves at the gas–liquid interfaces in the expanding dough.

These mechanisms of interactions between endogenous lipids and gluten proteins affect the viscoelastic properties of the gluten network under varying processing deformations. The following sections discuss the impact of endogenous wheat lipids on mixing, linear viscoelastic, and nonlinear viscoelastic properties of gluten proteins.

## 3 Impact of Endogenous Lipids on Hydration and Mechanical Network Development of Gluten Proteins

### 3.1 Hydration and Mechanical Network Development

**FIGURE 4:**
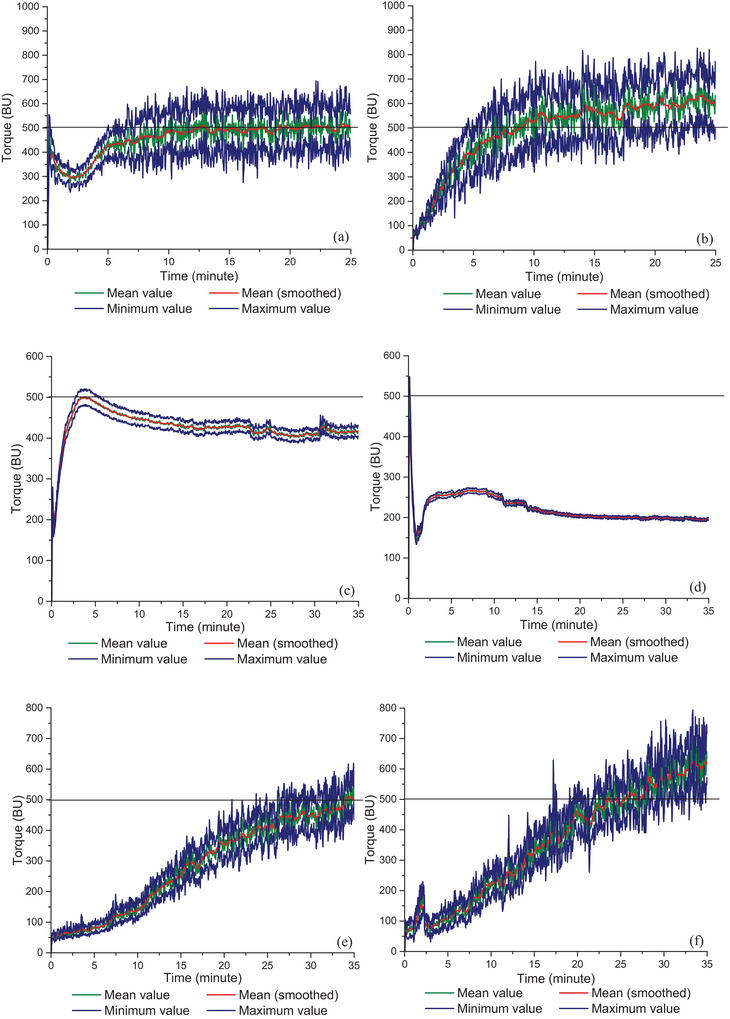
Farinograms for gluten, gliadin, and glutenin with and without endogenous lipids: (a) gluten, (b) lipid‐removed gluten, (c) gliadin, (d) lipid‐removed gliadin, (e) glutenin, and (f) lipid‐removed glutenin.

Farinograph mixing is a useful tool to investigate the mixing properties of gluten (Bushuk 1963; Yazar et al. 2022). Farinograph mixing for the gluten–water mixture showed a peak consistency followed by a gradual decrease in torque with continued mixing. Doguchi and Hlynka (1967) studied the mixing behavior of gluten extracted from hard red spring wheat flour. They obtained Farinograms similar to those observed for wheat flours. However, they encountered an initial difficulty in the mixing of wet gluten lumps. To eliminate this difficulty in homogeneous mixing of gluten at the beginning of Farinograph mixing, the application of a series of stop‐run mixing processes (for around 15 times) was used prior to conducting the complete Farinograph test (Doguchi and Hlynka 1967). Similarly, Yazar et al. (2022) reported an initial decay in consistency of gluten during the Farinograph mixing due to the difficulty in hydration as shown in Figure 4a, which was attributed to the presence of endogenous lipids (Yazar et al. 2022). Endogenous lipids decreased the affinity of gluten proteins to water by either shielding the gluten proteins or by forming gluten–lipid interactions leading to a decrease in the plasticization of gluten proteins by water (Papantoniou et al. 2004). The shielding of the positive charges in gluten proteins occurs through ionic bonds with the negatively charged lipids (Melis and Delcour 2020). As mixing started, the gluten powder turned into doughy large chunks with the introduction of water, and the endogenous lipids created a hydration blocking effect at this early stage of Farinograph mixing, which eventually led to a decrease in consistency as seen in Figure 4a (Yazar et al. 2022). The hydrocarbon chains between the two sides of a lipid bilayer in the lamellar structure (Figure 2), that are associated only by weak van der Waal's forces, break easily upon deformation to provide a slip‐plane with lubrication properties (Carr et al. 1992; Gerits et al. 2014). Ultimately, the doughy chunks observed during the early stages of gluten mixing by Yazar et al. (2022) were due to the partial deterioration of lamellar endogenous lipid structures under mixing forces.

Continued mixing enabled a better distribution of the lipids and overcame their blocking effect on the hydration of gluten proteins (Yazar et al. 2022). And the resulting gradual hydration of the gluten proteins with continued mixing resulted in an increase in consistency until reaching 500 BU as seen in Figure 4a. Once the 500 BU consistency was reached, the consistency of gluten remained constant until the end of mixing.

On the other hand, hydration of gluten proteins occurred gradually and more rapidly as endogenous lipids were extracted and removed (Figure 4b). With the same level of added water, gluten proteins with endogenous lipids reached the 500 BU consistency after 12 min of mixing (Figure 4a), whereas the same consistency was reached after a shorter 8 min of mixing (Figure 4b) in the absence of lipids (Yazar et al. 2022). The more rapid hydration of gluten proteins in the absence of endogenous lipids was due to the lack of interactions between lipids and the hydrophilic side chains of gluten proteins. Water access to gluten proteins occurred in an uncontrolled manner, because gluten proteins were not protected from water (Papantoniou et al. 2004). At the end of Farinograph mixing, the mean consistency for vital wheat gluten was around 500 BU (Figure 4a), whereas it became higher around 600 BU as endogenous lipids were removed (Figure 4b). A less elastic but more stable network was observed for gluten in the presence of endogenous lipids (Yazar et al. 2022). The interactions between proteins and lipids during mixing reduced the development of the protein matrix, leading to the formation of a softer network (Peng and Yao 2017).

Yazar, Kokini, et al. (2024) conducted Farinograph mixing tests on gliadin and glutenin both in the presence and absence of endogenous lipids to explore how endogenous lipids contribute to the mixing behaviors of the two main gluten protein fractions forming the gluten network. Gliadin with endogenous lipids showed a typical development peak (Figure 4c), that is commonly observed for wheat flours, after around 3 min of Farinograph mixing (Yazar, Kokini, et al. 2024; Yazar, Smith, et al. 2024). The consistency of gliadin started to decrease beyond the peak, then displayed an almost stabilized mixing curve throughout the rest of mixing (Figure 4c). When lipids were removed and the same level of water was given (Yazar, Kokini, et al. 2024), the consistency of gliadin decreased dramatically without showing peak formation (Figure 4d). The highest consistency obtained for the lipid‐removed gliadin was around 270 BU (Figure 4d), in comparison to the maximum consistency of 500 BU observed for gliadin (Figure 4c), indicating a reduction in the water absorption capacity of gliadin in the absence of endogenous lipids (Yazar, Kokini, et al. 2024).

Glutenin required more time to develop (34 min) in comparison to gliadin (3 min) and continued to display development throughout the whole mixing process without showing a peak (Figure 4e). Both with (Figure 4e) and without endogenous lipids (Figure 4f), the consistency of glutenin increased gradually as mixing proceeded. The consistency of 500 BU was reached more rapidly (23 min of mixing) when lipids were removed (Figure 4f). This was indicative of an increased water absorption capacity and a more rapid hydration for glutenin in the absence of endogenous lipids (Yazar, Kokini, et al. 2024). Table 1 shows how the Farinograph mixing parameters change for gluten, gliadin, and glutenin when endogenous lipids are present and absent.

**TABLE 1:** Farinograph mixing properties of gluten and the two main gluten fractions, gliadin and glutenin, with and without endogenous lipids.

Sample		Amount of water used for mixing (%)	Development time (min)	Consistency at development time (BU)	Maximum consistency (BU)
Native gluten and gluten fractions	Gluten^a^	65.2	≈4	≈700	≈700
	Gluten^b^	95 130	3–4 9–9.5	≈550 ≈300	≈550 ≈300
	Gluten^c^	118.8	12	500	500
	Gliadin^d^, ^e^	68	3.6	500	500
	Glutenin^d^, ^e^, ^f^	100.6	34	500	500
Gluten and gluten fractions without endogenous lipids	Lipid‐removed gluten^c^	118.8	8	500	603
	Lipid‐removed gliadin^d^	68	—	—	268
	Lipid‐removed glutenin^d^	100.6	23.2	500	617

^a^ Bushuk (1963).

^b^ Doguchi and Hlynka (1967).

^c^ Yazar et al. (2022).

^d^ Yazar, Kokini, et al. (2024).

^e^ Yazar, Smith, et al. (2024).

^f^ Yazar et al. (2023).

### 3.2 Molecular Interaction Mechanisms Associated With the Mixing Properties

**FIGURE 5:**
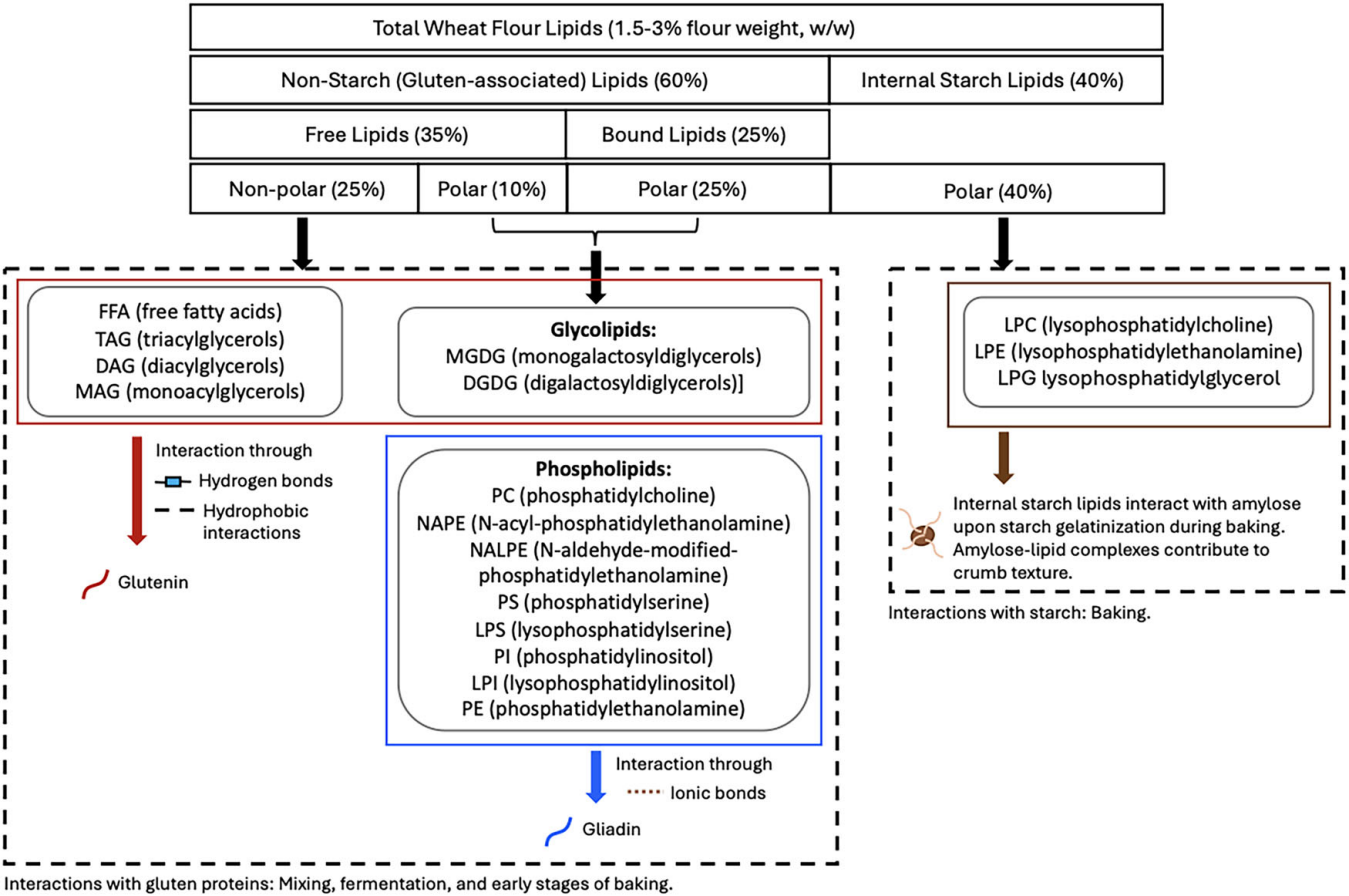
Classification of endogenous wheat flour lipids and their interactions with gluten proteins and starch during dough processing. Red, blue, and brown lines indicate the interactions with glutenin, gliadin, and starch, respectively.

Nonpolar lipids have been suggested to exist in lipid pockets encased within the gluten network, whereas polar lipids are considered to interact directly with the gluten proteins (McCann et al. 2009). The BLs in the gliadin fraction are known to be all polar, whereas glutenin proteins are bound to both polar and nonpolar lipids (Pareyt et al. 2011; Janssen et al. 2018). Gliadins and glutenins have the isoelectric points of 7.8 and 5.8, respectively (Lambrecht et al. 2017). In a freshly mixed dough, which has the pH of 5.8, gliadins are positively charged, whereas glutenins are neutral. Therefore, gliadins preferably interact with the zwitterionic (PC [phosphatidylcholine], LPC [lysophosphatidylcholine], PS [phosphatidylserine], and LPS [lysophosphatidylserine]) or negatively charged phospholipids (NAPE [*N*‐acyl‐phosphatidylethanolamine], NALPE [*N*‐aldehyde‐modified‐phosphatidylethanolamine], PI [phosphatidylinositol], LPI [lysophosphatidylinositol], PE [phosphatidylethanolamine], and LPE [lysophosphatidylethanolamine]) as shown in Figure 5. On the other hand, glutenins interact with the neutral glycolipids (MGDG [monogalactosyldiacylglycerols] and DGDG [digalactosyldiacylglycerol]) and nonpolar lipids (FFA, TAG, DAG (diacylglycerols), and MAG [monoacylglycerols]) (Figure 5). Gliadins and phospholipids interact through ionic bonds. Glutenin proteins interact with galactolipids via hydrophobic interactions and hydrogen bonding, whereas their interactions with nonpolar endogenous lipids occur through hydrophobic interactions (McCann et al. 2009; Pareyt et al. 2011; Janssen et al. 2018).

Phospholipids consist of a phosphate polar head‐group and a hydrophobic fatty acid tail, resulting in an amphiphilic molecule (Pichot et al. 2013). Gliadin molecules while primarily interacting with phospholipids, contribute significantly to an essential external hydrophobic trait (Monteiro et al. 2004). When endogenous lipids were removed, without the amphiphilic character of phospholipids, the ability of gliadin proteins to interact with water greatly diminished, as evidenced by the sharp decrease in the consistency of gliadin in the absence of lipids (Figure 4d). Similar to phospholipids, galactolipids are also known to display an amphiphilic nature (Pareyt et al. 2011). However, the impact of interactions with lipids on hydration properties of glutenins (Figure 4f) was the opposite of what had been observed for gliadins (Figure 4d). This difference is caused by the secondary structures of gliadin and glutenin upon hydration (Yazar, Kokini, et al. 2024), which is discussed in detail below.

**FIGURE 6:**
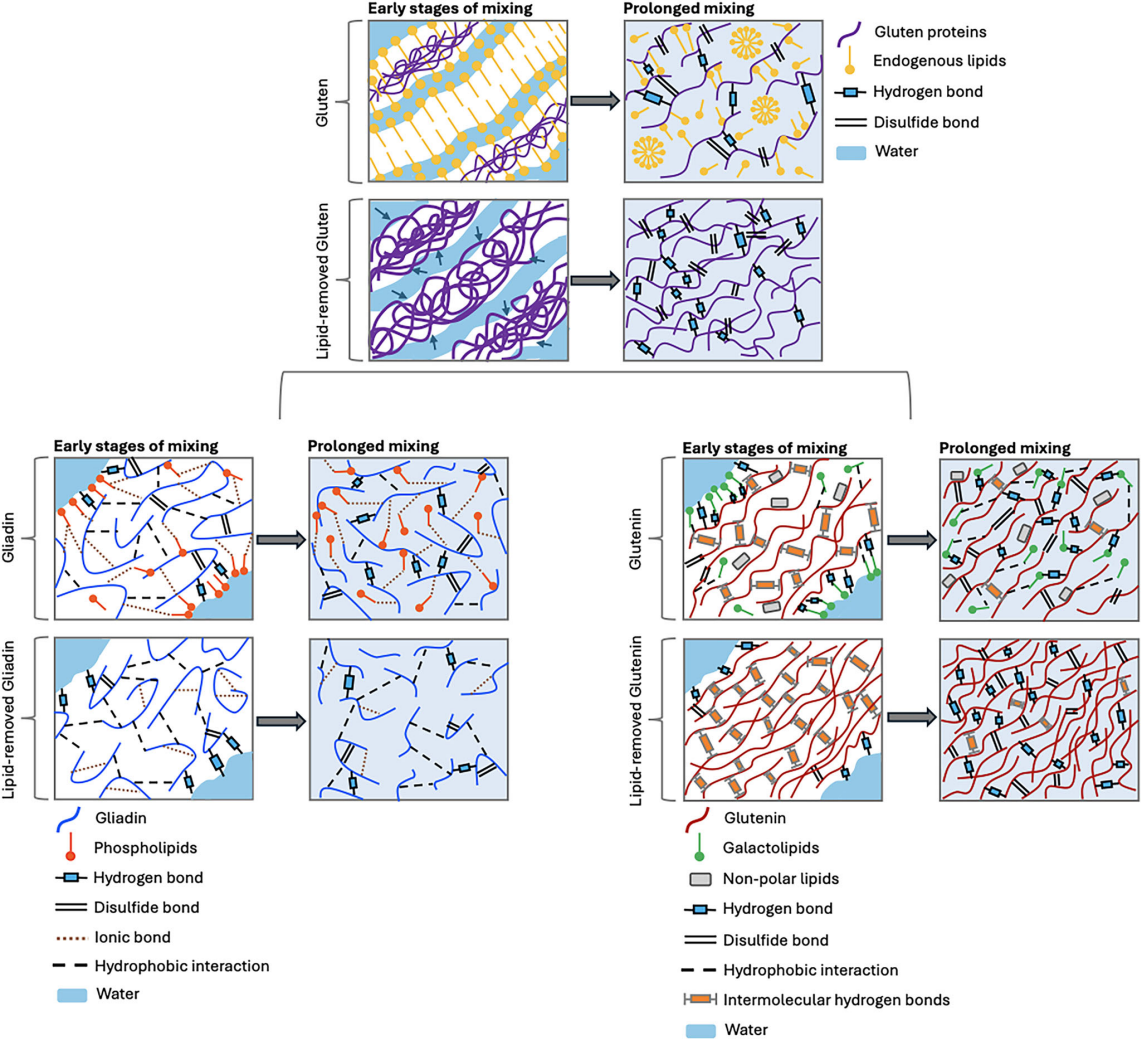
A zoomed‐in illustration of interactions among gluten proteins and endogenous lipids during mixing. These illustrations should be evaluated along with the Farinograms given in Figure 4. In the early stage of mixing, endogenous lipids form monolayers or liposomes that shield the gluten proteins or they interact with the gluten proteins, leading to a limited access of the gluten proteins to water (Papantoniou et al. 2004). As mixing continues, this shielding effect disappears and endogenous lipids enable the hydration of gluten proteins in a controlled manner. In the absence of endogenous lipids, the hydration of gluten proteins occurs faster. With continued mechanical deformation, more hydrophilic side chains of gluten proteins become available for interaction with water and hydration of the gluten proteins occurs in an uncontrolled manner, leading to a denser network (Yazar et al. 2022). Endogenous wheat lipids have the opposite effect on the mixing properties of gliadin and glutenin. Gliadins interact with phospholipids through ionic bonds (Janssen et al. 2018). The polar head groups of phospholipids align themselves towards water, and therefore, phospholipids act as bridges to convey water to gliadins. In the absence of endogenous lipids, hydration of gliadins becomes poorer. This results in more free water in the dough and decreases the consistency (Yazar, Kokini, et al. 2024). Glutenins interact with galactolipids through hydrogen bonds and hydrophobic interactions, while interacting with nonpolar lipids through hydrophobic interactions (McCann et al. 2009; Pareyt et al. 2011; Janssen et al. 2018). In the absence of endogenous lipids, the balance between the train (interchain hydrogen bonds between glutenins in the dry state) and the loop (glutenin–water hydrogen bonds) regions (see the train‐loop model proposed by Belton (1999)) is disrupted. The affinity of glutenin proteins to water increases with continued mixing, the loop regions dominate the system, and a denser network with higher consistency is obtained in the absence of lipids (Yazar, Kokini, et al. 2024).

The networks of gliadin, glutenin, and gluten in the presence and absence of endogenous lipids at the early stages and prolonged stages of the Farinograph mixing (Figure 4) are shown in Figure 6. As water meets gluten proteins in the early stages of mixing, the inter and intramolecular hydrogen bonds between the glutamine side chains and backbone peptide groups found in the dry state of gluten proteins are disrupted by water. The binding of water to gluten proteins occurs through hydrogen bonds preferentially at the polar peptide groups breaking the internal protein–protein hydrogen bonds (Wellner et al. 1996; Hernández‐Muñoz et al. 2004).

During the hydration stage of mixing, the interactions with phospholipids provide gliadins with polar sites to interact with water (Figure 6). Thus, phospholipids act as a bridge among gliadins and water and contribute to hydration (Figure 6). The gradual hydration increases the mobility of proteins that leads to rearrangements in their structure such as the formation of β‐sheets (Wellner et al. 1996; Hernández‐Muñoz et al. 2004). Until gluten proteins get fully hydrated, α‐gliadins and ω‐gliadins aggregate by hydrophobic interactions until the peak point is reached as seen in Figure 4c, and then they disintegrate with continued mixing (Iwaki et al. 2021; Li et al. 2021; Yazar, Smith, et al. 2024).

The β‐sheet structures formed in ω‐gliadins in the early stages of mixing are replaced with β‐turns, and extended structures are formed as hydration continued, indicating a decrease in protein‐protein interactions (Wellner et al. 1996; Shewry and Belton 2024). These results are consistent with the increase in the consistency of gliadin in the early stages of mixing, followed by a decrease in consistency as mixing continued (Figure 4c). In the absence of endogenous lipids, the number of polar sites decreases in gliadin due to the lack of phospholipids and thus, the matrix has more free water as seen in Figure 6. As mixing continued, protein molecules are stretched and aligned as they are subjected to shear and uniaxial extension deformations caused by the applied mechanical energy (Connelly and Kokini 2007), leading to dissociation of gliadin proteins (Figure 6) and a further decrease in consistency (Figure 4d). Besides the hydrophobic interactions occurring between α‐gliadins and ω‐gliadins, α/β‐ and γ‐gliadins are tied by covalent disulfide bonds and hydrogen bonds in their α‐helices and β‐sheets (Tatham and Shewry 1985). Although, hydrogen bonds are weaker than disulfide bonds, they play an important role in the rheological behavior of the gluten network by facilitating the reorientation of gluten proteins under stress (Ooms and Delcour 2019). The stabilization of gliadin's consistency under continued Farinograph mixing forces observed both in the presence and absence of endogenous lipids (Figure 4c,d) can be mainly attributed to the presence of hydrogen bonds (Yazar, Smith, et al. 2024). Ultimately, the most significant effect of gliadin–phospholipid interactions is increasing the hydration of gliadin proteins (Figure 6).

Fourier transform infrared spectroscopy (FTIR) showed that, unlike gliadin proteins, hydrated glutenin subunits primarily interact through stacked β‐sheets (Georget and Belton 2006; Shewry and Belton 2024). A significant increase in intermolecular β‐sheet structures over α‐helices has been observed as the ratio of glutenin to gliadin increased in hydrated gluten (Georget and Belton 2006). The increased levels of β‐sheet structures in wheat glutenin upon hydration are the result of the interactions between HMW glutenin subunits (Yazar et al. 2023). An increase in β‐sheet structures and extended structures has been found with the continued hydration of HMW glutenin (Dewan et al. 2022). This is consistent with the gradual development of glutenin up to 34 min of Farinograph mixing, as shown in Figure 4f. Belton (1999) proposed the train‐loop model to explain the high molecular weight protein mediated elastic properties of the gluten network. According to this model, the interchain hydrogen bonds existing between the protein chains in the dry state of HMW‐glutenins (trains) are replaced with the protein–water hydrogen bonds (loops) formed as HMW‐glutenins get hydrated, leading to an equilibrium between the train and the loop regions. The removal of endogenous lipids disrupts the balance between the formation of β‐sheet and the existing interchain hydrogen bonds during the mixing of glutenin (Yazar, Kokini, et al. 2024), which is evident from the progressive increase in the consistency of glutenin without endogenous lipids (Figure 4f). In the absence of interactions between lipids and the side chains of gluten proteins, water access to gluten proteins occurs in an abundant manner (Papantoniou et al. 2004). Farinograms reveal that glutenin proteins are not protected from water due to the lack of interactions with galactolipids and nonpolar lipids during mixing (Figure 4e,f). As mixing proceeds and glutenin proteins become more and more hydrated, the glutenin network displays a densely packed structure in the absence of endogenous lipids (Figure 6) due to the increase in the affinity of glutenin proteins to water.

As water is added to dry gluten, lipids shield the proteins at the very early stages as seen by the sudden drop in consistency (Figure 4a). Hydration occurs more slowly in the presence of lipids, indicating the hydration dynamics of the gluten network at the beginning of mixing are mostly governed by the interactions between endogenous lipids and glutenins (Figure 6). However, the aggregation of certain gliadin subunits (α‐gliadins and ω‐gliadins) and the interactions of gliadins with phospholipids bring gluten to the development point earlier than glutenin. As mixing proceeds, the presence of glutenins in the gluten network increases the consistency, whereas the dissociation of gliadins lowers the consistency, and the presence of endogenous lipids provides a balance to the consistency  of the protein matrix. Hydrogen bonds stabilizing α/β‐ and γ‐gliadins along with the hydrophobic forces that are brought on by endogenous lipids (mainly galactolipids and nonpolar lipids) to control the water affinity of glutenins collectively contribute to the stability of gluten. As a result, a more stable consistency is observed for the gluten network when endogenous lipids are present (Figure 4a). On the other hand, the consistency continues to increase when endogenous lipids are absent (Figure 4b), leading to a stiffer network (Figure 6).

## 4 Impact of Endogenous Wheat Flour Lipids on Linear Viscoelastic Properties of Gluten Proteins

Hargreaves et al. (1995) conducted SAOS measurements in the frequency range between *ω*: 0.001–36 Hz, *γ*: 3%, *T*: 20°C on gluten samples extracted from wheat flour samples treated with chloroform to remove endogenous lipids. Another batch was treated with lubrol to remove non‐gluten proteins to unravel the contribution of these minor wheat flour components to the SAOS behavior of gluten. Gluten proteins constitute around 80% of wheat proteins and they are responsible for the viscoelastic properties of wheat flour dough (Yazar 2023b). The remaining part of wheat proteins consists of soluble proteins such as albumins and globulins and insoluble proteins like membrane proteins, nucleoproteins, and cell wall proteins. Both soluble and insoluble non‐gluten protein fractions contain proteins displaying a high affinity to lipids that are known as lipid transfer proteins, puroindolines, ligoline, thionins, and so forth (Carr et al. 1992). The frequency sweeps conducted by Hargreaves et al. (1995) revealed no significant difference between the *G*′ and G″ values of gluten samples extracted from native, chloroform‐treated, and lubrol‐treated wheat flours, indicating that endogenous lipids and non‐gluten proteins did not contribute to the linear viscoelastic properties of gluten.

**FIGURE 7:**
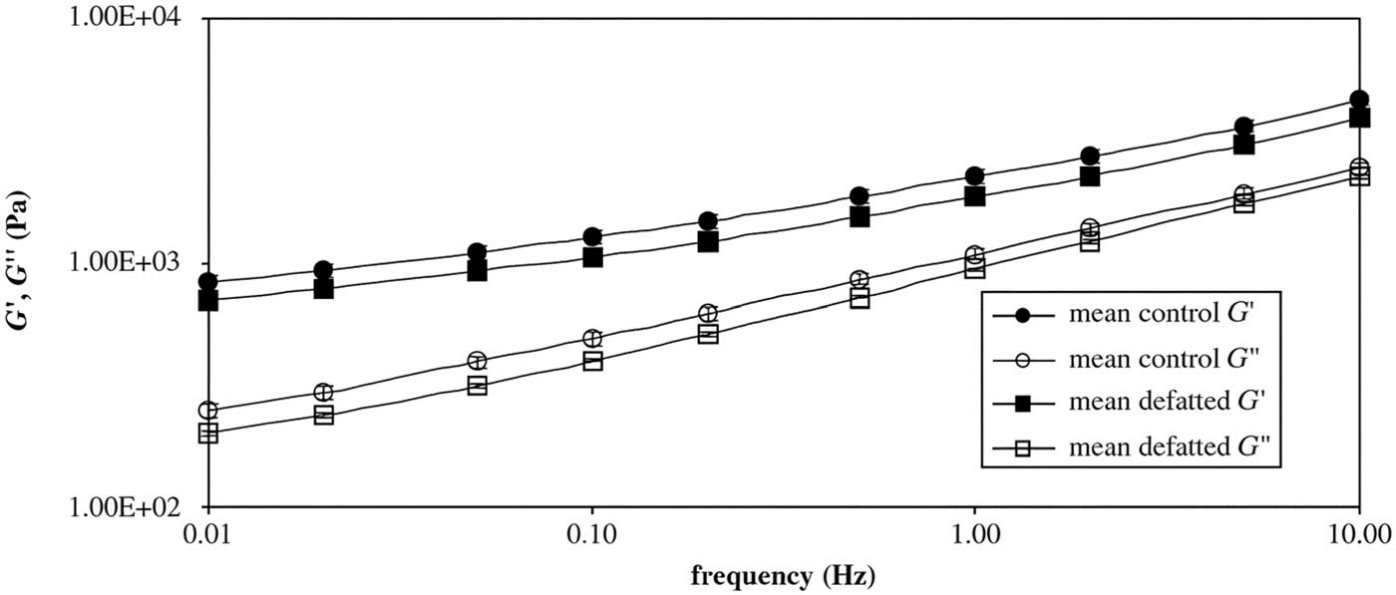
Frequency sweeps for gluten and defatted gluten. *Source*: Reproduced from Papantoniou et al. (2004) with the permission from the publisher.

Papantoniou et al. (2004) also studied the linear viscoelastic properties of gluten and defatted gluten. Defatted gluten displayed lower *G*′ and *G*″ in frequency sweeps in comparison to gluten containing endogenous lipids (Papantoniou et al. 2004) as shown in Figure 7. Georgopoulos et al. (2006), on the other hand, reported an increase in *G*′ versus frequency when endogenous lipids were removed (*ω*: 0.1–10 Hz, *T*: 25°C).

Different gluten extraction methods can also lead to differences in the linear viscoelastic properties (Hargreaves et al. 1995; Papantoniou et al. 2004; Georgopoulos et al. 2006), causing differences in the moisture contents of the native and lipid‐removed gluten samples. In the experimental settings of Papantoniou et al. (2004) and Georgopoulos et al. (2006), the extracted wet gluten samples were directly measured without being dried. The water contents of defatted gluten and native gluten studied by Papantoniou et al. (2004) were 68.8% and 65%, respectively. The higher level of water absorption in the defatted gluten was attributed to the higher affinity of gluten to water in the absence of endogenous lipids (Papantoniou et al. 2004; Rocca‐Smith et al. 2016; Yazar et al. 2022), which caused lower *G*′ (*ω*) for the lipid‐removed gluten. In contrast, Georgopoulos et al. (2006) reported the water contents of 55.4% for native gluten and 53.8% for defatted gluten, leading to a higher *G*′ (*ω*) for the defatted gluten in comparison to native gluten. These results unraveled the importance of selecting the appropriate rheological testing protocol and the importance of adjusting the water contents of both native and lipid‐removed glutens to the same level prior to rheological testing to accurately determine the impact of endogenous lipids on viscoelastic properties.

**FIGURE 8:**
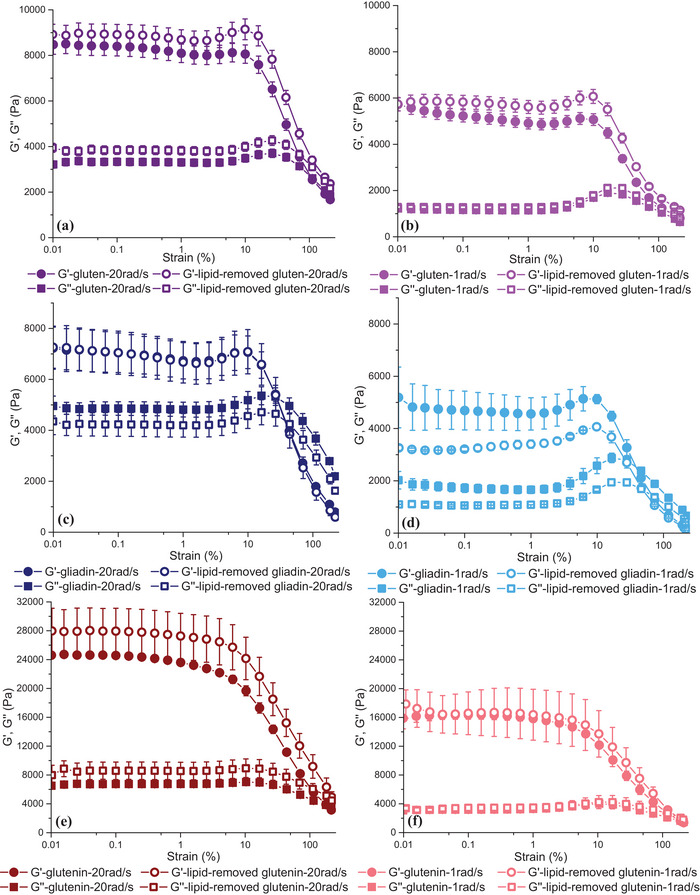
Strain sweeps for vital wheat gluten and lipid‐removed vital wheat gluten (a and b), gliadin and lipid‐removed gliadin (c and d), and glutenin and lipid‐removed glutenin (e and f) at high (20 rad/s) and low (1 rad/s) frequencies. Data at high frequency were shown in darker shades, whereas those at low frequency were given in light shades.

To eliminate the issues arising due to the different water contents of gluten and defatted gluten in capturing the actual impact of endogenous lipids on the viscoelastic nature of gluten network, Yazar et al. (2022) studied the rheological properties of vital wheat gluten and lipid‐removed vital wheat gluten with the same level of added water. Yazar et al. (2022) removed endogenous lipids directly from gluten instead of wheat flour. This allowed capturing the contribution of endogenous lipids to the gluten network strength without the interference of other wheat flour components. Both vital gluten and lipid‐removed gluten in dry states were mixed in a Farinograph mixer with the same level of added water. Their SAOS properties were studied. The *G*′ values of gluten obtained in the linear viscoelastic region were not significantly affected by the removal of endogenous lipids. However, the lower phase angle (*δ*) values reported for gluten with endogenous lipids at high frequencies revealed the contribution of endogenous lipids to the networking ability of gluten (Yazar et al. 2022). The interactions between endogenous lipids and gluten were shown to contribute to the strength of the gluten network by promoting the interaction of hydrophobic amino acids (Gerits et al. 2013). This effect of endogenous lipids seemed to disappear at low frequencies as evidenced by the similar strain sweep data obtained in the linear viscoelastic region for gluten (Figure 8a) and lipid‐removed gluten (Figure 8b).

The rheological approach used by Yazar et al. (2022) to determine the effect of endogenous lipids on the viscoelastic properties of hydrated gluten under small deformations was taken one step further and applied on the main gluten fractions, gliadin and glutenin (Yazar, Kokini, et al. 2024).

The linear viscoelastic properties of hydrated gluten fractions and their lipid‐removed counterparts were determined. At low frequency, removal of lipids resulted in a notable decrease in *G*′ values of gliadin (Figure 8d). However, this effect disappeared when the frequency was high (Figure 8c). Besides, *G*″ values of gliadin showed a decrease at all frequencies when endogenous lipids were removed.

The analysis of the loss tangent (tan *δ* = *G*″/*G*′) revealed no significant difference upon lipid removal, which suggested that endogenous lipids did not affect the viscoelastic network properties of gliadin under small deformations. Unlike gliadin, removal of endogenous lipids caused an increase in both *G*′ and *G*″ values of glutenin, but only at high frequency (Figure 8e). *G*′ and *G*″ values of glutenin and lipid‐removed glutenin were almost similar at low frequency (Figure 8f). Therefore, endogenous lipids did not affect the tan *δ* of glutenin. Even though, the increasing *G*′ and *G*″ data obtained at high frequency for glutenin upon the removal of lipids suggested a stiffer structure for glutenin in the absence of lipids, tan *δ* values were significantly higher for the lipid‐removed glutenin, suggesting an increase in the viscous to elastic ratio of the glutenin viscoelastic network when endogenous lipids were removed.

Ultimately, the strain sweep data obtained by Yazar, Kokini, et al. (2024) in the linear viscoelastic region revealed no notable contribution of endogenous lipids to the viscoelastic behavior of gliadin, while pointing to a more rigid structure with reduced networking ability for glutenin in the absence of lipids that became evident only at high frequencies.

**FIGURE 9:**
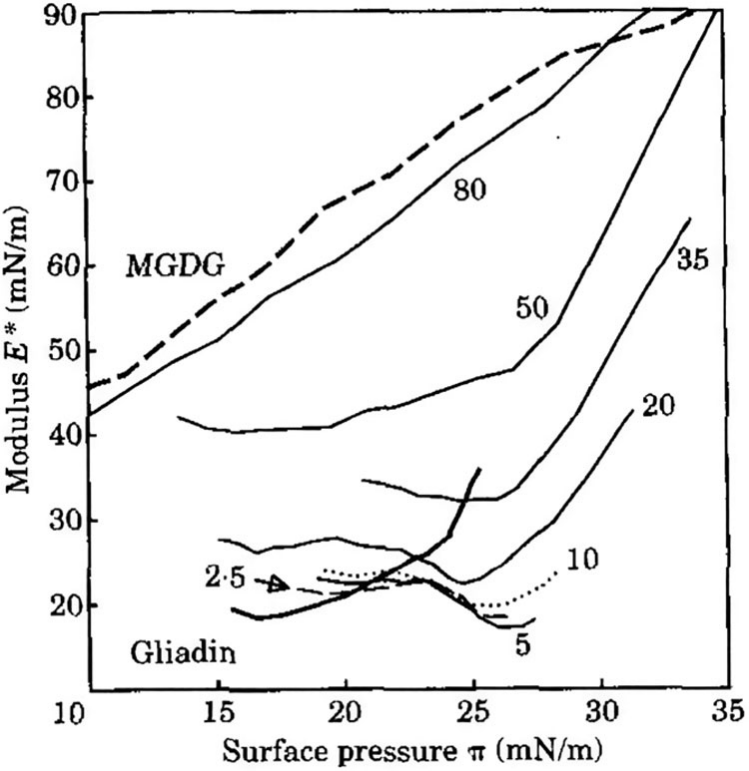
Film rigidity evaluation for gliadin, MGDG, and gliadin‐MGDG films. The compositions of the films were given as percent MGDG dry weight. *Source*: Reproduced from Paternotte et al. (1994) with the permission from the publisher. MGDG, monogalactosyldiacylglycerols.

Paternotte et al. (1994) studied the interfacial behavior of defatted gliadin films with added MGDG to explore the contribution of gliadin in the presence of a polar lipid to gas cell stability in wheat flour dough. MGDG are among the non‐starch polar lipids found in wheat flour (Pareyt et al. 2011; Gerits et al. 2013). Polar lipids like MGDG orient themselves strongly at the gas–liquid interface as condensed monolayers. These condensed monolayers generate the required elastic storing energy, which resist destabilization of the liquid lamellae depicted in Figure 3b when changes in interfacial area occur with continued dough expansion (Sroan and MacRitchie 2009). The dilational measurement results obtained for defatted gliadin‐MGDG films with different gliadin to MGDG ratios pointed to a reduction in film rigidity for the films containing MGDG in the range of 2.5%–35% (w:w), as evidenced by the decrease in the surface dilational modulus (E*) as a function of increasing surface pressure (*π*) up to 25 mN/m (Figure 9). This decay in E* was not observed for the pure defatted gliadin film and the defatted gliadin films with MGDG above 35% (w:w), indicating that the addition of small amounts of MGDG into gliadin film prevented gliadins from forming a coherent protein network throughout the film and thus caused the film to be unstable (Paternotte et al. 1994).

**FIGURE 10:**
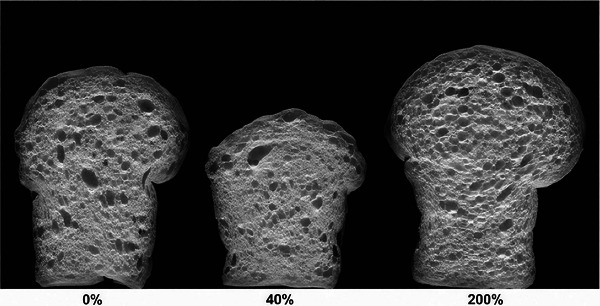
Effect of endogenous wheat polar lipids on loaf volume when added at different levels to defatted wheat flour as percentage of endogenous flour lipids. *Source*: Reproduced from Sroan and MacRitchie (2009) with the permission from the publisher.

Similarly, a minimum in loaf volume was reported for defatted wheat flour bread with the addition of native flour polar lipids at small percentages (Sroan and MacRitchie 2009) as shown in Figure 10. These results indicated that endogenous lipids contributed to the strain‐stiffening behavior of the gluten network, as the strain‐stiffening behavior of the gluten–starch matrix has been associated with the loaf volume of the resulting bread (Figure 3b). An optimum strain‐stiffening value for doughs was reported for an improved loaf volume. When the strain‐stiffening behavior of a dough system was above or below the optimum, a decrease was recorded in the loaf volume of the resulting bread (Yazar et al. 2017a).

The defatted gliadin displayed a collapse above the surface pressure of 25 mN/m due to gliadin proteins being completely folded, whereas the pure MGDG film was able to resist much higher surface pressures (Paternotte et al. 1994) as shown in Figure 9.

Kokelaar and Prins (1995) reported an increase in dilational complex modulus, E* values of wheat endogenous lipids as their concentration increased from 5 to 12 mg/m^2^. Beyond the concentration of 12 mg/m^2^, E* gradually decreased. At lipid concentrations ranging from 10 to 12 mg/m^2^, a sudden increase in the loss tangent of the surface was found, indicating a sharp shift from elastic to viscous behavior. This was attributed to the formation of multiple lipid layers or micelles from which the lipids can diffuse towards the surface, leading to a smaller change in surface tension during deformation of the surface (Kokelaar and Prins 1995). In defatted flour, proteins are the only surface‐active compounds to form monolayers with high surface elasticity. The presence of lipids at the liquid–gas interface results in the formation of mixed monolayers and reduces the elasticity of the monolayers at the gas–liquid interfaces (Salt et al. 2006; Sroan and MacRitchie 2009).

Wouters et al. (2019) evaluated the impact of endogenous lipids on the interfacial rheology of gliadin using oscillating pendant drop measurements. Dilational elastic (*E*′) and viscous (*E″*) moduli of defatted gliadin nanoparticles showed a more pronounced increase versus time in comparison to those of native gliadin nanoparticles, indicating an increased film rigidity in the absence of endogenous lipids. This result confirmed that gliadin proteins and lipids interact to form a viscoelastic film at the air–water interface and suggested that endogenous lipids soften the adsorbed protein film at the air–water interface (Wouters et al. 2019). The lower *E*′ and *E″* values obtained for gliadin nanoparticles with endogenous lipids (Wouters et al. 2019) agreed with the lower E* obtained for the defatted gliadin film with added MGDG at low concentrations (Paternotte et al. 1994). These dilational properties of gliadin and lipid films suggested that lipids were responsible for imparting the desired extensibility to the gliadin–lipid film against the disproportionation of the gas cell surface occurring during gas cell expansion. However, it would not be completely accurate to make such a conclusion based on the data provided by Paternotte et al. (1994) and Wouters et al. (2019), as interfacial properties of gliadin films in these studies were evaluated under small oscillation amplitudes, whereas dough expansion involves large deformations (Menjivar 1990). Therefore, the next section highlights the effect of endogenous wheat lipids on the nonlinear viscoelastic properties of gluten, gliadin, and glutenin for a better understanding of how gluten–lipid interactions contribute to the viscoelastic nature of the gluten network under large deformations similar to those experienced during dough processing. Table 2 brings a comparison of how endogenous lipids affect the viscoelastic properties of the gluten network and the individual viscoelastic properties of gliadin and glutenin under small and large deformations.

**TABLE 2:** Comparison of linear and nonlinear viscoelastic responses of gluten, gliadin, and glutenin in the presence and absence of endogenous lipids.

Sample	Effect of removal of endogenous lipids	
	Rheological method: Linear viscoelastic response	Rheological method: Non‐linear viscoelastic response
Gluten	Frequency sweep: *G*′ and *G*″ values were not affected^a^ Frequency sweep: *G*′ and *G*″ values decreased^b^ Frequency sweep: *G*′ values increased^c^ Strain sweep: *G*′ values were not affected. Phase angle values increased at 20 rad/s, but they were not affected at 1 rad/s^d^	LAOS tests: Phase angle values decreased. A counterclockwise rotation of the elastic Lissajous–Bowditch curves and an increase in the total stress response were observed. Viscous Lissajous–Bowditch curves became wider. The tendency to type IV nonlinear behavior increased^d^
Gliadin	Strain sweep: *G*′ and *G*″ values decreased at 1 rad/s. However, tan *δ* values were not affected at both high (20 rad/s) and low (1 rad/s) frequencies^e^ Oscillating pendant drop: *E*′ and *E″* values increased^f^	LAOS tests: A clockwise rotation of the elastic Lissajous–Bowditch curves and a decrease in the total stress response were observed. Viscous Lissajous–Bowditch curves became slightly narrower^e^
Glutenin	Strain sweep: *G*′, *G*″, and tan *δ* values were not affected at 1 rad/s. At 20 rad/s, *G*′ and *G*″ did not change notably; however, tan *δ* values increased significantly^e^	LAOS tests: A counterclockwise rotation of the elastic Lissajous–Bowditch curves and an increase in the total stress response were observed. Viscous Lissajous–Bowditch curves became wider. These changes were more pronounced under high‐frequency deformations^e^

^a^ Hargreaves et al. (1995).

^b^ Papantoniou et al. (2004).

^c^ Georgopoulos et al. (2006).

^d^ Yazar et al. (2022).

^e^ Yazar, Kokini, et al. (2024).

^f^ Wouters et al. (2019).

## 5 Impact of Endogenous Wheat Flour Lipids on Nonlinear Viscoelastic Properties of Gluten Proteins

**FIGURE 11:**
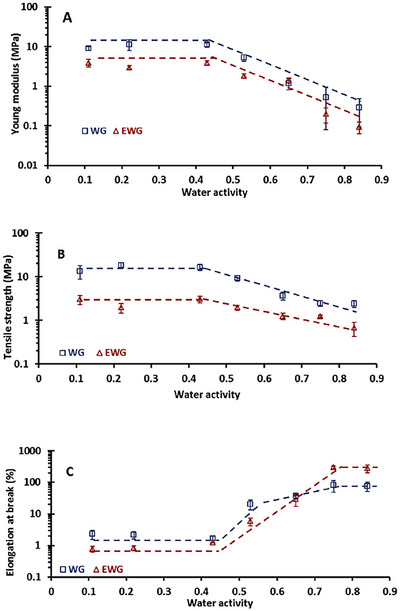
Mechanical properties of WG (wheat gluten films) and EWG (emulsified wheat gluten films with the added lipid phase) as a function of water activity at 25°C: (a) Young's modulus, (a) tensile strength, and (c) elongation at break. *Source*: Reproduced from Rocca‐Smith et al. (2016) with the permission from the publisher.

Gluten films with and without added lipid, consisting of a commercial blend of acetic acid esters of mono and diglycerides and beeswax with glycerol monostearate as emulsifier, were fabricated. The mechanical properties of these films were studied using uniaxial tensile tests (Rocca‐Smith et al. 2016) and the effects of the different compositional parameters were compared. Although this study did not evaluate the impact of endogenous wheat lipids on mechanical properties of gluten films, the acetic esters of mono and diglycerides in the lipid blend used by Rocca‐Smith et al. (2016) were also part of wheat endogenous lipids. MAGs and DAGs are among the nonpolar lipids, and nonpolar lipids constitute around 33.2%–47.4% of non‐starch (gluten‐associated) lipids (Melis and Delcour 2020). These acylglycerols make up a portion of phospholipids and galactolipids found in wheat flour (Janssen et al. 2018). The uniaxial tensile tests revealed reductions both in the Young modulus (Figure 11a) and tensile strength (Figure 11b) of gluten films with the addition of the lipid phase as a function of water activity. The decrease in these parameters as a function of water activity was associated with the lower cohesion in the gluten network, arising from the formation of hydrophobic interactions with added lipids. These hydrophobic interactions hinder the intermolecular bonds between gluten proteins, causing discontinuities in the gluten network under large deformations (Rocca‐Smith et al. 2016).

A higher elongation at break for the gluten film with the added lipid was observed in comparison to that without lipids as the water activity increased beyond 0.75 (Figure 11c) (Rocca‐Smith et al. 2016). The amphiphilic nature of some lipids acted as lubricants enabling the sliding of molecular chains and thus leading to the formation of a softer and a more extensible gluten network (Rocca‐Smith et al. 2016). These results explained the lower *G*′ values reported for gluten through the strain sweeps in comparison to those obtained for the lipid‐removed gluten in the nonlinear viscoelastic region (Figure 6a,b) (Yazar et al. 2022).

At strains above 4%, gluten starts to display nonlinear viscoelastic behavior (Wang and Kokini 1995; Lefebvre et al. 2003). The strain sweeps conducted on gluten and lipid‐removed gluten showed a similar critical strain (*γ*_cri_) values of around 3%–4%, as seen in Figure 8a,b. Critical strain (*γ*_cri_) is a strain amplitude value determined in dynamic oscillatory shear tests, at which the transformation from linear to nonlinear viscoelastic region occurs (Erturk et al. 2023). The strain sweep data in Figure 8a,b suggested that the removal of endogenous lipids did not influence the onset of nonlinearity for the gluten network (Yazar et al. 2022). Similarly, the strain sweeps were also conducted on gliadin, glutenin, and their lipid‐removed counterparts (Yazar, Kokini, et al. 2024). *G*′ and *G*″ obtained in the LAOS sweeps indicated a *γ*_cri_ of 1.5% for gliadin and lipid‐removed gliadin, while revealing a *γ*_cri_ of 4% for glutenin and lipid‐removed glutenin at 10 rad/s. A more resilient network for glutenin in comparison to gliadin both in the presence and absence of endogenous lipids was found as the applied strain amplitude gradually increased (Yazar, Kokini, et al. 2024). Other studies also reported an extended linear viscoelastic region for glutenin when compared to gliadin (Yazar et al. 2017b; Yazar, Smith, et al. 2024). None of these studies evaluated the impact of endogenous lipids on the nonlinear viscoelastic responses of gluten proteins. As *G*′ and *G*″ in the strain sweeps did not capture any differences in the *γ*_cri_ values of gliadin and glutenin, Yazar, Kokini, et al. (2024) used the harmonic intensities (*I*) for an in‐depth evaluation of the *γ*_cri_ for the gluten proteins with and without endogenous lipids. In the linear viscoelastic region, a material's stress response is determined by the first harmonic extracted from the LAOS tests via Fourier analysis. As the amplitude of strain gradually increases and the material transitions into the nonlinear region, the stress response deviates from the ideal sinusoidal pattern and higher odd‐number harmonics appear along with the first harmonic in the Fourier transform spectrum (Erturk et al. 2023). At the onset of nonlinearity, which is characterized as the Medium Amplitude Oscillatory Shear (MAOS) region, the third harmonic's intensity constitutes the only higher harmonic contribution, and it is an indicator for the onset of nonlinear viscoelastic behavior. It also enables defining the boundaries of the MAOS region (Ewoldt and Bharadwaj 2013; Singh et al. 2018; Song and Hyun 2019). The measurement of the harmonic intensities yields a more precise identification of the critical strain amplitude for the transition from SAOS to MAOS regions (Erturk et al. 2023). The *γ*_cri_ values obtained for the low‐strain boundaries of the MAOS region (*I*_2_/*I*_3_<0) were 2.5% and 4% for gliadin and lipid‐removed gliadin, respectively (Yazar, Kokini, et al. 2024). Unlike *G*′ and *G*″, harmonic intensities revealed an increase in the *γ*_cri_ of gliadin with the removal of endogenous lipids. On the other hand, *I*_2_/*I*_3_ revealed a shift in the *γ*_cri_ of glutenin from 4% to 2.5% as endogenous lipids were removed (Yazar, Kokini, et al. 2024). Harmonic intensities revealed that the removal of endogenous lipids affected the viscoelastic responses of gliadin and glutenin in an adverse manner at the onset of nonlinear viscoelastic region.

**FIGURE 12:**
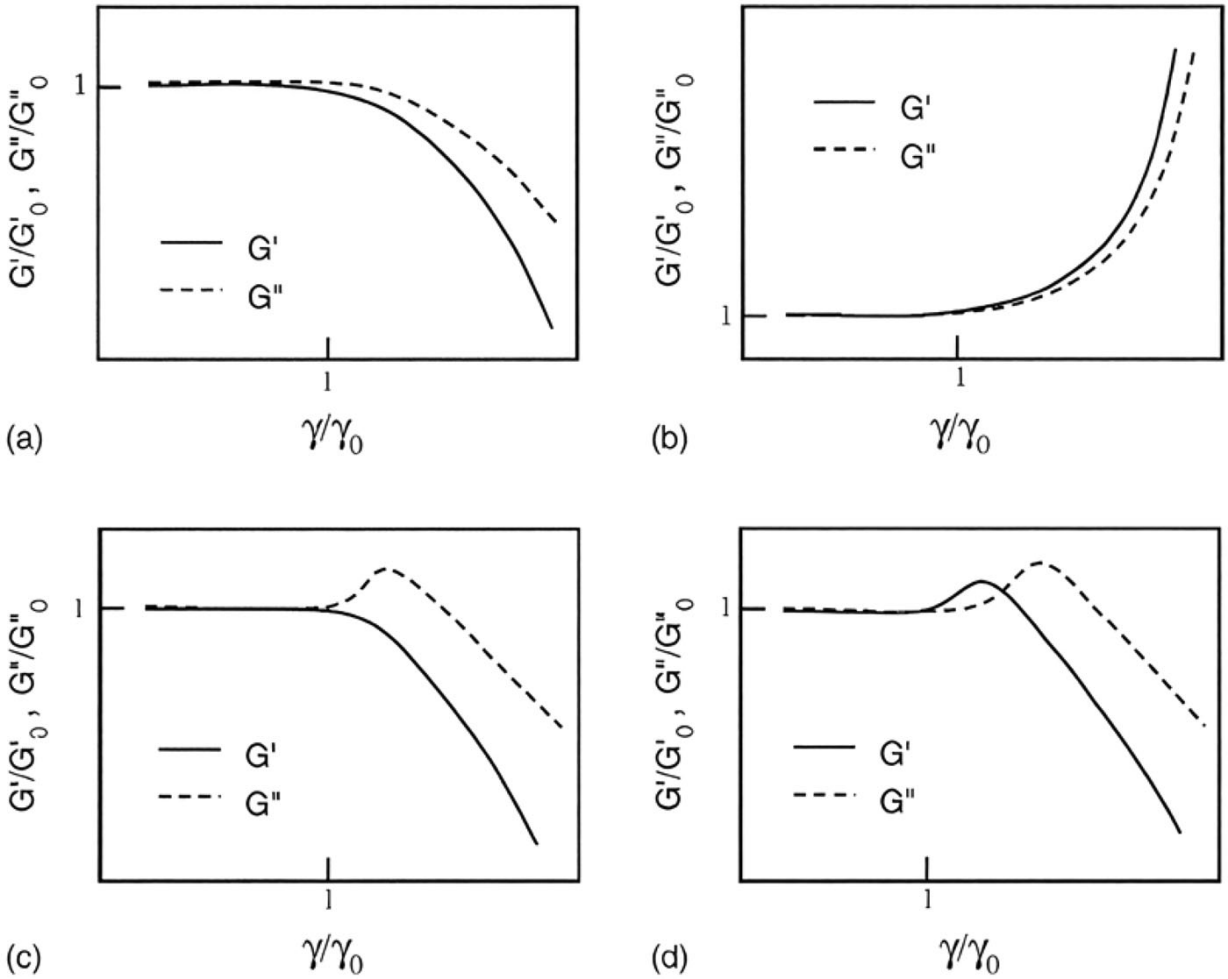
The types of LAOS behavior: (a) type I—strain thinning; (b) type II—strain hardening; (c) type III—weak strain overshoot; (d) type IV—strong strain overshoot. Subscript 0 indicates a value selected in the linear viscoelastic region. *Source*: Reproduced from Hyun et al. (2002) with permission from the publisher.

The evaluation of the first harmonic material functions, that are indicators of intercycle elastic (*G*′) and viscous (*G*″) properties, in the MAOS region has led to the classification of different types of LAOS behaviors (Hyun et al. 2002), as shown in Figure 12. In the linear viscoelastic region, polymer chains are found in an entangled state and therefore *G*′ and *G*″ remain constant (Figure 12). As the amplitude of strain continued to increase, polymer chains either disentangle and align with the flow field, causing a decrease in the moduli (intercycle strain‐softening and shear‐thinning), or they further entangle and lead to an increase in the moduli (intercycle strain‐stiffening and shear‐thickening) (Hyun et al. 2002; Duvarci et al. 2019).

**FIGURE 13:**
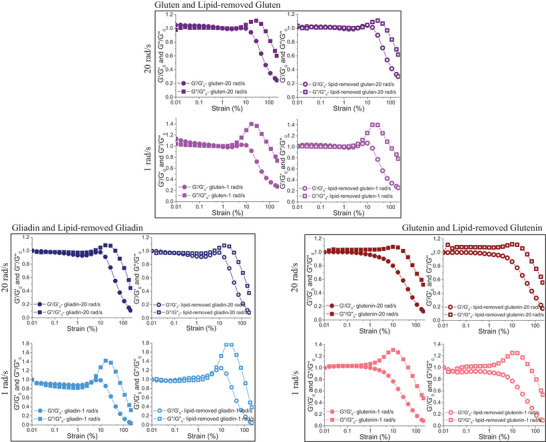
Reduced moduli for gluten, gliadin, glutenin, and their lipid‐removed counterparts.

According to the classification brought by Hyun et al. (2002), glutenin showed type III (weak strain overshoot) LAOS behavior (Yazar et al. 2017b). It displayed an overshoot in *G*″ accompanied with a decrease in *G*′ throughout the nonlinear viscoelastic region (Figure 13). The degree of overshoot in *G*″ was not affected by the removal of endogenous lipids, indicating a type III nonlinear behavior for glutenin also in the absence of lipids (Yazar, Kokini, et al. 2024).

On the other hand, gliadin showed type IV nonlinear behavior (Yazar et al. 2017b), as evidenced by the *G*′ and *G*″ overshoots observed in the LAOS sweeps (Figure 13). The extent of the overshoots increased as endogenous lipids were removed from gliadin, especially when the deformation frequency was low. Nevertheless, the type of LAOS behavior gliadin displayed did not change in the absence of endogenous lipids (Yazar, Kokini, et al. 2024). Ultimately, gluten showed a combination of type III and type IV nonlinear behaviors (Yazar et al. 2022). The removal of endogenous lipids from gluten caused an increase in the extent of *G*′ overshoot (Yazar et al. 2022), leading to a nonlinear behavior approximating type IV for gluten in the absence of lipids (Figure 13). This indicated an increase in the intercycle strain‐stiffening behavior of the gluten network caused by the higher degree of entanglements in the absence of endogenous lipids.

In type III nonlinear behavior, when the material is exposed to an external deformation, the complex structure resists against deformation up to a certain strain amplitude, where *G*″ increases. As the amplitude of strain continued to increase beyond the critical strain, the polymer chains align with the flow field and *G*″ decreases (Figure 12) that lead to the disruption of the complex structure by large deformations (Hyun et al. 2002). In type IV nonlinear behavior, the origin of *G*′ and *G*″ overshoots at intermediate strain amplitudes (Figure 12) is caused by intermolecular interactions between the hydrophobic groups in a network structure.

The interaction energy in networks displaying type IV nonlinear behavior is considered to be stronger than those with type III nonlinear behavior, where only *G*″ shows an overshoot (Hyun et al. 2002). These definitions of LAOS behavior types on a macromolecular basis reveal that gliadin had a higher ability to withstand the increasing strain amplitudes in the MAOS region in comparison to glutenin (Figure 13), which could be attributed to gliadin's extensible nature (Wieser 2007; Shewry and Belton 2024).

The removal of endogenous lipids from gliadin did not change the extent of the resistance against the increasing strain amplitudes in the MAOS region when the deformation frequency was high. On the other hand, the removal of lipids caused gliadin to display a higher degree of resistance with increasing deformations at low frequency, causing greater intercycle strain‐stiffening and shear‐thickening behaviors. Glutenin was less resistant against the deformation when transitioning into the nonlinear region in comparison to gliadin (Figure 13) (Uthayakumaran et al. 2000). Thus, glutenin was more brittle in the MAOS region.

The removal of endogenous lipids did not cause any significant change in glutenin's intercycle strain‐softening and shear‐thickening responses at the onset of nonlinearity at both high and low frequencies (Figure 13). These results showed that gliadin was mainly responsible for the *G*″ overshoot in gluten under moderate strain amplitudes. The slightly higher *G*″ overshoot observed for gluten when endogenous lipids were absent seems to be due to the lack of gliadin–lipid interactions. In the absence of lipids, gluten displayed intercycle shear‐thickening to a higher extent. Glutenin, on the other hand, played a significant role in stabilizing the intercycle strain‐stiffening behavior both in the presence and absence of endogenous lipids (Figure 13). Ultimately, glutenin–lipid interactions do not contribute to the intercycle behavior of gluten under moderate deformations.

**FIGURE 14:**
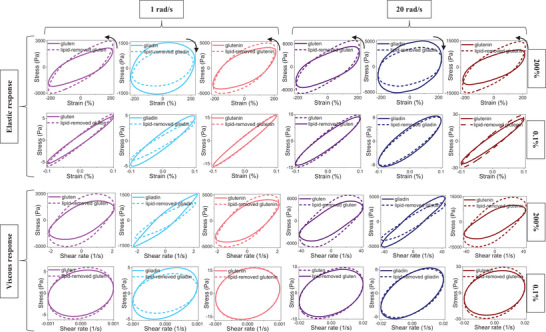
Raw elastic and viscous Lissajous–Bowditch curves for gluten, gliadin, glutenin, and their lipid‐removed counterparts in the SAOS (at 0.1% strain) and LAOS (at 200% strain) regions at low (1 rad/s) and high (20 rad/s) frequencies.

Gluten and lipid‐removed gluten samples hydrated with the same quantity of water showed similar elliptical trajectories in the elastic Lissajous–Bowditch curves at small strain amplitudes (Figure 14), indicating similar viscoelastic behaviors in the linear region. This result was in line with the strain sweep data for gluten and lipid‐removed gluten shown in Figure 8. The impact of endogenous lipids on nonlinear viscoelasticity became apparent at large strain amplitudes. Elastic Lissajous–Bowditch curves displayed counterclockwise rotation in the absence of lipids (Figure 14), which is associated with an increase in elasticity (Ewoldt et al. 2007). Besides, the higher stress magnitudes observed for both gluten and lipid‐removed gluten at 20 rad/s frequency in comparison to 1 rad/s (Figure 14) revealed a magnified effect of endogenous lipids on increasing the elasticity at high frequencies. The wider viscous Lissajous–Bowditch curves (Figure 14) obtained for the lipid‐removed gluten under large deformations (*γ*: 200%) supported the increase in elasticity (Yazar et al. 2022).

Strain sweep tests were also conducted on the two main gluten fractions, gliadin and glutenin, and their lipid‐removed counterparts to bring more detailed insights into the interactions among endogenous lipids and gluten proteins and the resulting impacts on the individual viscoelastic properties of these protein fractions that form the gluten network (Yazar, Kokini, et al. 2024). Elastic and viscous Lissajous–Bowditch curves obtained for glutenin and lipid‐removed glutenin in the linear viscoelastic region (*γ*: 0.1%) displayed similar trajectories (Figure 14), indicating no significant effect of endogenous lipids on the linear viscoelastic behavior of glutenin under small deformations. However, elastic Lissajous–Bowditch curves of lipid‐removed gliadin at small strains and at low frequency showed a clockwise rotation in comparison to those of gliadin (Figure 14). This result revealed an elastic decay for gliadin in the absence of lipids under conditions similar to those experienced during the resting stages of dough processing. This can be regarded as an indication of how the interactions between gliadins and endogenous lipids contribute to stabilization of the viscous flow properties of dough during resting. The degree of flow depends on the ratio between viscous and elastic properties (Uthayakumaran et al. 2000; Delcour and Hoseney 2010). A higher degree of viscous flow would disrupt the mechanical properties of dough, leading to poor dough handling (Spies 1990).

In the nonlinear viscoelastic region (*γ*: 200%), the removal of endogenous lipids resulted in opposite viscoelastic behavior for gliadin and glutenin (Yazar, Kokini, et al. 2024). In the absence of lipids, the Lissajous–Bowditch curves of gliadin displayed clockwise rotation (Figure 14), indicating softening for gliadin under large deformations. The Lissajous–Bowditch curves of glutenin, on the other hand, showed counterclockwise rotation as lipids were removed (Figure 14), pointing to a stiffening behavior in the absence of lipids at large strain amplitudes.

The Lissajous–Bowditch curves obtained in the nonlinear region were consistent with the Farinograph data (Figure 4). Thus, both the Farinograms and the Lissajous–Bowditch curves unraveled the fact that gliadin–lipid–glutenin interactions provided the gluten network with a balance in viscous to elastic responses under large deformations.

In the nonlinear region, distortions from the native trajectories were observed both for elastic and viscous Lissajous–Bowditch curves (Figure 14). These distortions that appear as abnormal bending in the Lissajous–Bowditch curves were further evaluated through the Chebyshev coefficients to show the impact of lipids on the nonlinear intracycle behaviors of gluten (Yazar et al. 2022) and its main fractions (Yazar, Kokini, et al. 2024). The emergence of third‐order harmonics has been associated with the presence of nonlinearity. The first‐order harmonic material functions give information regarding the intercycle (global) behaviors, whereas the third‐order material functions indicate the intracycle (local) deviation from linearity within a single oscillation cycle (Ewoldt and Bharadwaj 2013). Therefore, the ratios of the third‐order elastic (*e*_3_) and viscous (*v*_3_) Chebyshev coefficients to the first‐order Chebyshev coefficients (*e*_1_, *v*_1_) were evaluated for gluten, gliadin, and glutenin proteins with and without endogenous lipids to reveal the impact of endogenous lipids on the intracycle nonlinear responses of gluten proteins.

**FIGURE 15:**
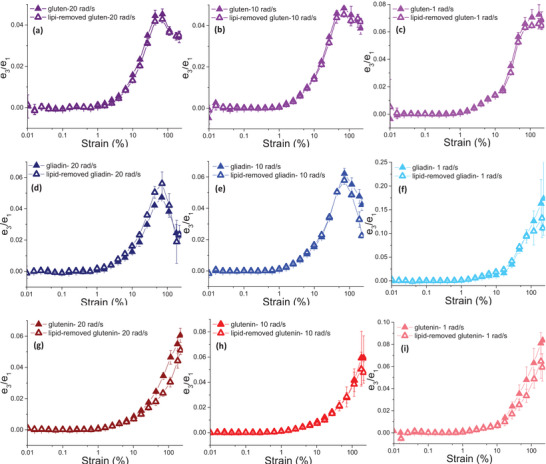
*e*_3_/*e*_1_ values for gluten (a–c), gliadin (d–f), glutenin (g–i), and their lipid‐removed counterparts as a measure of intracycle strain‐stiffening behavior.

Removal of endogenous lipids from gliadin resulted in an increase in *e*_3_/*e*_1_ values under moderate deformations at high frequency, indicating a higher degree of intracycle strain‐stiffening behavior. This effect disappeared as the strain amplitude continued to increase (Figure 15d). However, *e*_3_/*e*_1_ values started to decrease with the removal of endogenous lipids from gliadin under large strain amplitudes as the deformation frequency decreased to 10 rad/s (Figure 15e) and 1 rad/s (Figure 15f). Ultimately, elastic Chebyshev coefficients pointed to the contribution of endogenous lipids to the intracycle strain‐stiffening behavior of gliadin under moderate (*γ*: 25%–70%) to large (*γ* > 100%) deformations depending on the frequency, which could not be precisely captured through the elastic Lissajous–Bowditch curves (Yazar, Kokini, et al. 2024).

The *e*_3_/*e*_1_ values of glutenin were found to decrease with the removal of endogenous lipids only under large strain amplitudes at 20 rad/s (Figure 15), suggesting a lower degree of intracycle strain‐stiffening behavior for glutenin under large deformations with high frequency, as lipids were removed.

Unlike the impact of endogenous lipids on the intracycle response of gliadin, endogenous lipids had no significant effect on the intracycle strain‐stiffening behavior of glutenin at large strain amplitudes with low frequency (Yazar, Kokini, et al. 2024). No significant difference was found for *e*_3_/*e*_1_ of gluten as endogenous lipids were removed (Figure 15a–c).

The interactions among gliadin and glutenin in gluten have more pronounced influence on the intracycle strain‐stiffening response of gluten rather than the interactions between endogenous lipids and gluten proteins. However, the effect of endogenous lipids on the individual strain‐stiffening responses of gliadin and glutenin could play an important role during dough fermentation depending on how these proteins position themselves within the gluten–starch matrix. As mentioned in the earlier sections, the surface‐active compounds of wheat flour, such as polar lipids and gliadin proteins, stabilize the gas cells during dough expansion (Figure 3b) by aligning themselves at the gas cell interface in the gluten–starch matrix (Sroan and MacRitchie 2009). On the other hand, glutenin proteins form the skeleton of the gluten–starch matrix, which is responsible for gas cell stabilization (Sroan et al. 2009; Yazar et al. 2017b). Dough experiences large deformations during fermentation and oven‐rise due to yeast activity. The magnitudes of these deformations gradually increase as the yeast continues to produce CO_2(g)_, which in turn increases the pressure in the gas cells (Menjivar 1990). The frequency of proofing deformations is lower than those experienced during oven‐rise, as dough expansion occurs at lower temperatures over a longer time range during proofing in comparison to oven‐rise. Therefore, dough undergoes relatively moderate deformations during fermentation. The rapid expansion occurring during oven‐rise involves much larger deformations (Menjivar 1990; Yazar, Kokini, et al. 2024). As shown through the *e*_3_/*e*_1_ values, endogenous lipids altered the strain‐stiffening response of gliadin under relatively moderate to large deformations based on the frequency (Figure 15d–f). The strain‐stiffening behavior of glutenin proteins was affected by lipids at relatively large and rapid deformations (Figure 15g–i). Therefore, the interactions occurring between gliadin and endogenous lipids during dough expansion contributed to loaf volume both during proofing and oven‐rise. On the other hand, the effect of glutenin–lipid interactions on the strain‐stiffening behavior of glutenin proteins seems to occur mainly during oven‐rise where the dough expansion rate is much faster compared to proofing.

**FIGURE 16:**
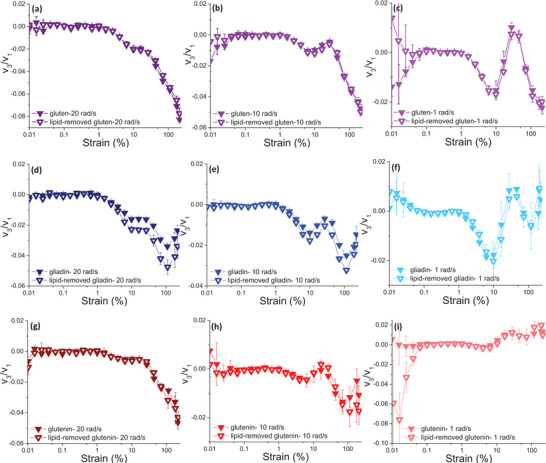
*v*_3_/*v*_1_ values for gluten (a–c), gliadin (d–f), glutenin (g–i), and their lipid‐removed counterparts as a measure of intracycle shear‐thinning/‐thickening behavior.

Viscous Chebyshev coefficients indicated intracycle shear‐thinning for gliadin in the nonlinear viscoelastic region (Yazar, Kokini, et al. 2024), as *v*_3_/*v*_1_ values were <0 (Ewoldt et al. 2008). Shear‐thinning behavior occurs as the polymer chains align themselves in the direction of flow (Hyun et al. 2002; Duvarci et al. 2019). Under large strain amplitudes, the removal of endogenous lipids from gliadin resulted in a decrease in the intracycle shear‐thinning (Figure 16d,e). When the frequency was low, the effect of lipids on the intracycle shear‐thinning behavior of gliadin disappeared (Yazar, Kokini, et al. 2024). Both gliadin and lipid‐removed gliadin changed signs at large strain amplitudes (*v*_3_/*v*_1_>0) showing intracycle shear‐thickening behavior (Figure 16f). The intracycle nonlinear response of glutenin was mainly shear‐thinning (*v*_3_/*v*_1_<0) (Figure 16g,h) and completely transitioned into shear‐thickening (*v*_3_/*v*_1_>0) behavior as the frequency decreased (Figure 16i). The *v*_3_/*v*_1_ values for glutenin both in the presence and absence of lipids indicated no significant effect of endogenous lipids on the intracycle shear‐thinning/‐thickening response of glutenin under large deformations, as evidenced by the similar *v*_3_/*v*_1_ values (Yazar, Kokini, et al. 2024). Even though, endogenous lipids were found to influence the individual intracycle viscous response of gliadin, when gliadin and glutenin interact to form the gluten network, the similar *v*_3_/*v*_1_ values found for both gluten and lipid‐removed gluten (Figure 16a–c) suggested no significant effect of endogenous lipids on the intracycle shear‐thinning/‐thickening behavior.

On the other hand, the magnitudes of the *v*_3_/*v*_1_ for gluten were relatively higher when compared to those of gliadin and glutenin, indicating a higher extent of intracycle shear‐thinning behavior for the gluten network, especially at large strain amplitudes. These *v*_3_/*v*_1_ values showed that the interactions between gliadin and glutenin in the gluten network had a greater influence on the intracycle shear‐thinning/‐thickening response of the gluten network than the interactions occurring between endogenous lipids and gluten proteins.

All these rheology data obtained for gluten proteins with and without endogenous lipids suggest that the gluten network development occurs in the presence of gliadin, glutenin, and water under mechanical forces. The minor wheat constituents like endogenous lipids provide continuity and resilience to the gluten network and improve its ability to better withstand the processing deformations.

## 6 Conclusions

Although endogenous lipids constitute a minor fraction in wheat flour, its contribution to the rheological properties of wheat flour dough is often neglected. This review provided an in‐depth evaluation on how endogenous lipids could affect the mechanical properties of gluten proteins forming the gluten network and should not be neglected.

Mixing studies showed a sudden drop in gluten's consistency at the early stages of mixing, caused by the gluten proteins being shielded by endogenous lipids. The hydration of gluten occurred more slowly when lipids were present. The interactions among gliadin and the phospholipid component of endogenous lipids contributed to the hydration and the stability of the gluten network. During the mechanical development stage of mixing, the interactions between glutenins and endogenous lipids consisting mainly galactolipids and nonpolar lipids seemed to improve the stability of the gluten network by controlling the water affinity of glutenins.

Studies focusing on the impact of endogenous wheat lipids on the linear viscoelastic properties of the gluten network revealed conflicting results. This was due to the different hydration protocols applied in these studies to prepare the gluten doughs for rheological testing. When gluten and defatted gluten samples were hydrated using the same amount of water, small deformation tests revealed no significant change in *G*′ and *G*″ but pointed to an increase in phase angle at high frequencies with the removal of endogenous lipids. This review showed that endogenous lipids contributed in a significant way to the strength of the gluten network. Removal of endogenous lipids from glutenin caused a similar effect. However, *G*′ and *G*″ of gliadin decreased under small deformations when endogenous lipids were removed. Endogenous lipids appear to control the extent of the viscous flow during dough resting.

Under large deformations, gliadin and glutenin showed the opposite responses with the removal of lipids. This shows that the simultaneous interactions among gliadin, glutenin, and endogenous lipids provide the gluten network with a balanced nonlinear viscoelastic response. When intracycle nonlinear responses were evaluated, endogenous lipids were found to affect the elastic measures rather than the viscous properties. On the basis of the elastic Chebyshev constants, it was hypothesized that the interactions between gliadin and endogenous lipids as part of liquid lamella contributed to loaf volume by manipulating the strain‐stiffening behavior of gliadins during proofing and oven‐rise, whereas glutenin–lipid interactions affected the strain‐stiffening behavior of glutenins mainly during oven‐rise.

The insights obtained through the discussions in this review will improve our fundamental understanding of the mechanisms behind the protein–lipid interactions in wheat flour dough and how these interactions contribute to dough rheology and baked product quality.

### 6.1 Future Perspectives and Practical Applications

‐ The genotype and environmental factors, including soil type, climate conditions, and agronomic applications such as the use of nitrogen fertilizers and clipping, influence the composition of wheat. Future studies may focus on this topic from an agricultural standpoint to reveal the impact of such growing conditions on the lipid content and composition of wheats.
‐ Wheat breeders and growers may benefit from the fundamental data provided in this review to better select for and refine desired characteristics in wheat. Therefore, future studies may involve developing wheat varieties with end product‐specific lipid compositions and gluten quantity/quality.
‐ The compositional differences in wheat flours of different wheat varieties lead to differences in the gluten network formation during dough mixing and to differences in the viscoelastic properties of wheat flour dough under processing deformations. This, in return, will result in baked products with different quality characteristics. Well‐characterized wheat varieties from different types (soft, hard, and durum) can be evaluated in future studies to explore the impact of varietal differences on gluten–lipid interactions and the resulting effects on dough rheology. This would provide millers with another parameter to consider while blending wheat to obtain flour with a more precise end product‐specific quality.
‐ This review focused on endogenous non‐starch wheat lipids while evaluating the impact of gluten–lipid interactions on the mechanical properties of the gluten network. And it covered rheological characterization at ambient temperature. Future studies may focus on the use of fundamental rheological tests conducted at high temperatures resembling the conditions of baking. It should also be noted that starch lipids are involved in interactions with starch during baking. Therefore, it would be interesting for future studies to evaluate the interactions of both starch and non‐starch endogenous wheat lipids with gluten and starch through thermal and rheological analyses coupled with the baking tests.
‐ The main limitation associated with studying the impact of endogenous wheat lipids on the viscoelastic properties of gluten and/or individual gluten fractions is the need for large quantities of samples. This leads to working with crude gluten fractions, and nevertheless, excessive extraction work is still required. Therefore, novel techniques that enable in situ evaluation of these interactions before, during, and after rheological testing would provide deeper insights.
‐ Understanding thoroughly how endogenous wheat lipids affect the mechanical properties of the gluten network and wheat flour dough will open new paths for future studies to explore the impact of external lipids or fat replacers on the mechanical properties of doughs and the resulting baked product quality.

## Author Contributions

**Gamze Yazar**: conceptualization, writing – original draft, writing – review and editing, investigation, visualization. **Jozef L. Kokini**: conceptualization, investigation, writing – review and editing, visualization, validation.

## Conflicts of Interest

The authors declare no conflicts of interest.
